# *De novo* chromosome 7q36.1q36.2 triplication in a child with developmental delay, growth failure, distinctive facial features, and multiple congenital anomalies: a case report

**DOI:** 10.1186/s12881-017-0482-8

**Published:** 2017-10-23

**Authors:** Muna A. Al Dhaibani, Diane Allingham-Hawkins, Ayman W. El-Hattab

**Affiliations:** 1 Pediatrics Department, Tawam Hospital, Al-Ain, United Arab Emirates; 2 PreventionGenetics, LLC, Marshfield, WI USA; 3 Division of Clinical Genetics and Metabolic Disorders, Pediatric Department, Tawam Hospital, Al-Ain, United Arab Emirates

**Keywords:** Chromosome 7q36, Genomic rearrangements, Chromosomal microarray, Chromosomal disorders

## Abstract

**Background:**

Studying human genome using chromosomal microarrays has significantly improved the accuracy and yield of diagnosing genomic disorders. Chromosome 7q36 deletions and duplications are rare genomic disorders that have been reported in a limited number of children with developmental delay, growth retardation, and congenital malformation. Altered dosage of *SHH* and *HLXB9*, both located in 7q36.3, is believed to play roles in the phenotypes associated with these rearrangements. In this report we describe a child with 7q36.1q36.2 triplication that is proximal to the 7q36.3 region. In addition to the clinical description, we discuss the genes located in the triplicated region.

**Case presentation:**

We report a 22 month old male child with a *de novo* 1.35 Mb triplication at 7q36.1q36.2. His prenatal course was complicated by oligohydramnios, intrauterine growth restriction, and decreased fetal movement. Hypotonia, respiratory distress, and feeding difficulty were observed in the neonatal period. He also had developmental delay, cardiovascular malformation, growth failure with microcephaly, short stature, and underweight, sensorineural hearing loss, myopia, astigmatism, cryptorchidism, hypospadias, microphallus, lower extremity length discrepancy, bifid uvula, single palmer creases, and distinctive facial features including straight eyebrows, ptosis, up-slanted palpebral fissures, broad nasal bridge, low-set and posteriorly rotated ears, small mouth with thick lower lip, microretrognathia, and high-arched palate.

**Conclusions:**

The child presented here had developmental delay, distinctive facial features, multiple congenital anomalies, and 7q36.1q36.2 triplication. This triplication, which was found to be *de novo*, has not been previously described and is believed to result in the observed phenotype. The triplicated region harbors the *GALNTL5*, *GALNT11*, *KMT2C*, *XRCC2*, and *ACTR3B* genes. *GALNT11* encodes a membrane-bound polypeptide N-acetylgalactosaminyltransferase that can O-glycosylate NOTCH1 leading to the activation of the Notch signaling pathway. Therefore, increased *GALNT11* dosage can potentially alter the Notch signaling pathway explaining the pathogenicity of 7q36 triplication. Studying further cases with similar genomic rearrangements is needed to make final conclusions about the pathogenicity of this triplication.

## Background

Studying human genome using chromosomal microarrays has significantly improved the accuracy and yield of diagnosing genomic disorders. Therefore, chromosomal microarrays have been routinely used as a first-tier diagnostic test for individuals with neurodevelopmental disabilities or congenital anomalies [1]. Compared to the 3% diagnostic yield of karyotyping, chromosomal microarray can detect pathogenic copy number variations (CNVs) with a diagnostic yield of 10-20% [2, 3].

Chromosome 7q36 rearrangements are rare genomic disorders. To date, deletions of 7q36 have been described in 47 individuals with developmental delay and growth retardation. Other common features in these individuals are microcephaly, ear malformation, cleft lip and palate, holoprosencephaly, and sacral malformation. Haploinsufficiency of the genes *SHH* and *HLXB9*, both located in 7q36.3, are believed to be responsible for the holoprosencephaly and sacral malformation, respectively [4]. Duplications involving the 7q36.3 region have been less frequently reported. Four individuals from a three-generation family were reported with 7q36.3 duplication and intellectual disability, corpus callosum agenesis, Chiari malformation, macrocephaly, and distinctive facial features [5]. Additionally, 7q36.3 duplications were reported in two siblings with muscle hypertrophy and an infant with encephalocele [6, 7]. Although *SHH* overexpression has been proposed to be responsible for the observed phenotypes in individuals with 7q36.3 duplications, the reason for heterogeneity in the phenotypes remains unclear [5].

In this report we describe a 7q36.1q36.2 triplication that is proximal to the 7q36.3 region and does not include *SHH* in a child with developmental delay, growth failure, distinctive facial features, and multiple congenital anomalies.

## Case presentation

A 22 months male child born to a second degree cousins who were healthy and had three healthy daughters. He was born at 35 week gestational age via a Cesarean section due to fetal distress. During pregnancy the mother had gestational diabetes that was diet controlled. The prenatal course was complicated by oligohydramnios, intrauterine growth restriction (IUGR), and decreased fetal movement noticed since the beginning of the third trimester. After birth, he was kept in neonatal intensive care unit (NICU) because of low birth weight (1.6 kg) and respiratory distress that required oxygen by nasal cannula for 2 weeks. He also had feeding difficulties required nasogastric tube feeding during the hospitalization. He was discharged home after 6 weeks when his oral feeding and respiratory distress improved.

He also had bilateral cryptorchidism, hypospadias, shallow scrotum, and microphallus. At the age of 3 months the stretched penile length was 1.7 cm. At that age, sonography showed testes in inguinal canals, karyotype showed 46 XY, and hormonal studies showed normal testosterone, luteinizing hormone (LH), thyroid function test, and insulin-like growth factor 1 (IGF-1). He received monthly testosterone injections for 3 months and the stretched penile length increased to 3.8 cm at the age of 6 months. At the age of 1 year he had bilateral orchidopexy.

Bilateral sensorineural hearing loss was diagnosed at the age of 13 months when he had an auditory brainstem response (ABR) testing. Subsequently, he started using hearing aids. In addition, he had snoring and otorhinolaryngology examination showed adenoid hypertrophy. Therefore bilateral myrigotomy and adenoidectomy were performed at the age of 18 months. In addition, he had bilateral congenital ptosis and eye exam at the age of 1 year revealed myopia and astigmatism.

During the NICU stay an echocardiogram showed patent ductus arteriosus with left to right shunt, branch pulmonary artery narrowing, and small aortic arch distal to the brachiocephalic artery and descending aorta. During the first year of life, he had several episodes of respiratory distress that required three hospitalizations and he was diagnosed to have reactive airway disease.

He started talking when he was 20 months and at the time of this report he could say about 10 words. He started rolling over at age of 7 months, sitting unsupported at 8 months, standing holding objects at 18 months, and at the time of this report he was able to walk holding furniture. He could also grasp and move objects from hand to hand, and waive bye-bye. He also had hypotonia since neonatal period. He started receiving speech therapy and physical therapy since the age of 1 year, with which he showed good response with improvements in hypotonia and his motor and language development. Brain MRI was done at the age of 20 months and was normal.

He also had growth failure with all growth parameters below 5th percentiles. He had been eating regular table food and had normal swallowing assessment at the age of 1 year.

**Fig. 1:**
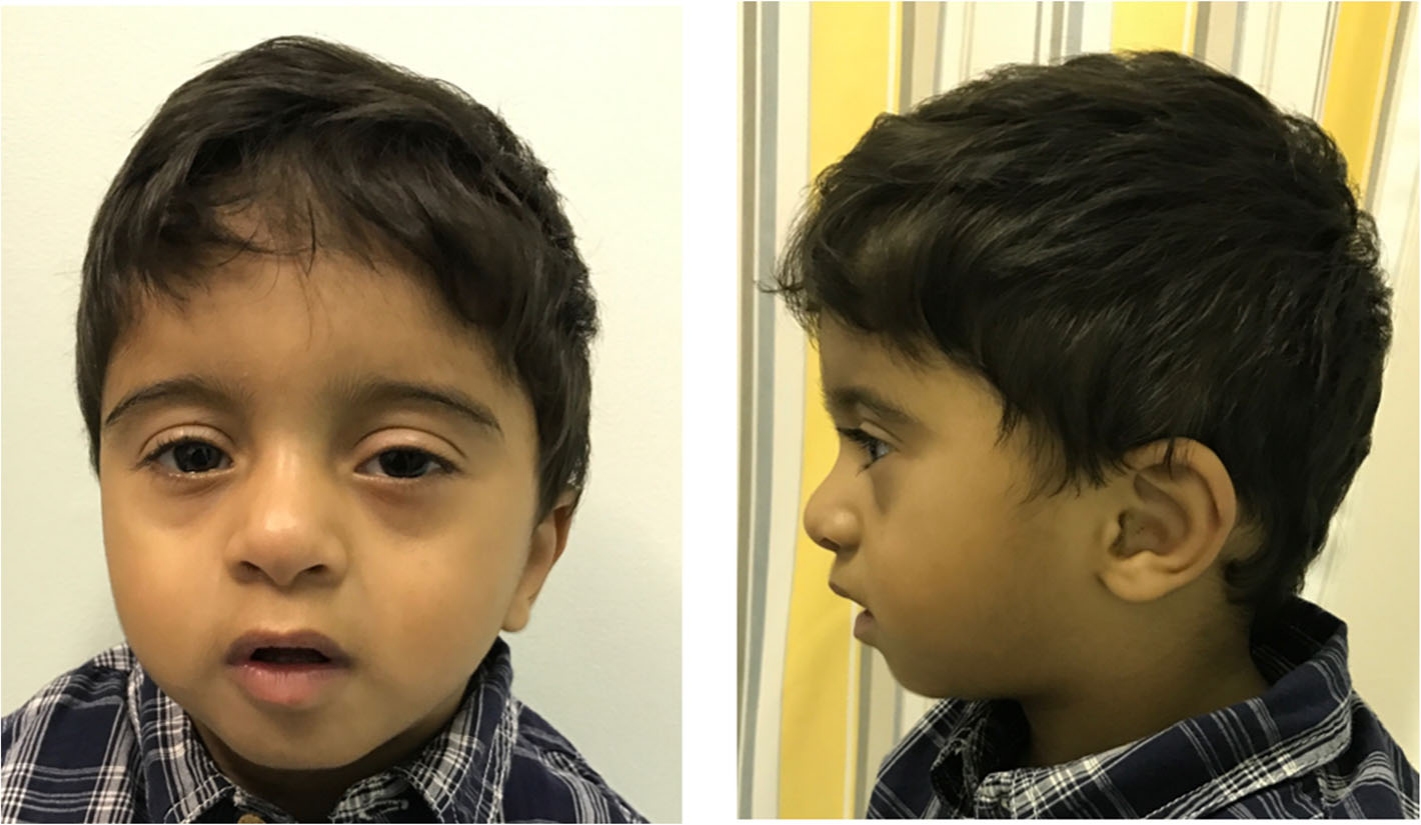
Photograph showing the following distinctive facial features: straight eyebrows, bilateral ptosis with the left side being more pronounced than the right, up-slanted palpebral fissures, broad nasal bridge, low-set and posteriorly rotated ears, small mouth, thick lower lip, and microretrognathia

On physical examination his weight was 9.2 kg, length 77 cm, and head circumference 44.5 cm (all at about 3 SD below means). Distinctive facial features were observed including: straight eyebrows, bilateral ptosis with the left side being more pronounced than the right, up-slanted palpebral fissures, broad nasal bridge, low-set and posteriorly rotated ears, small mouth with thick lower lip, microretrognathia, and high-arched palate with bifid uvula (Fig. 1). Single palmer crease was observed bilaterally and right lower extremity was longer than the left by 1 cm.

**Fig. 2:**
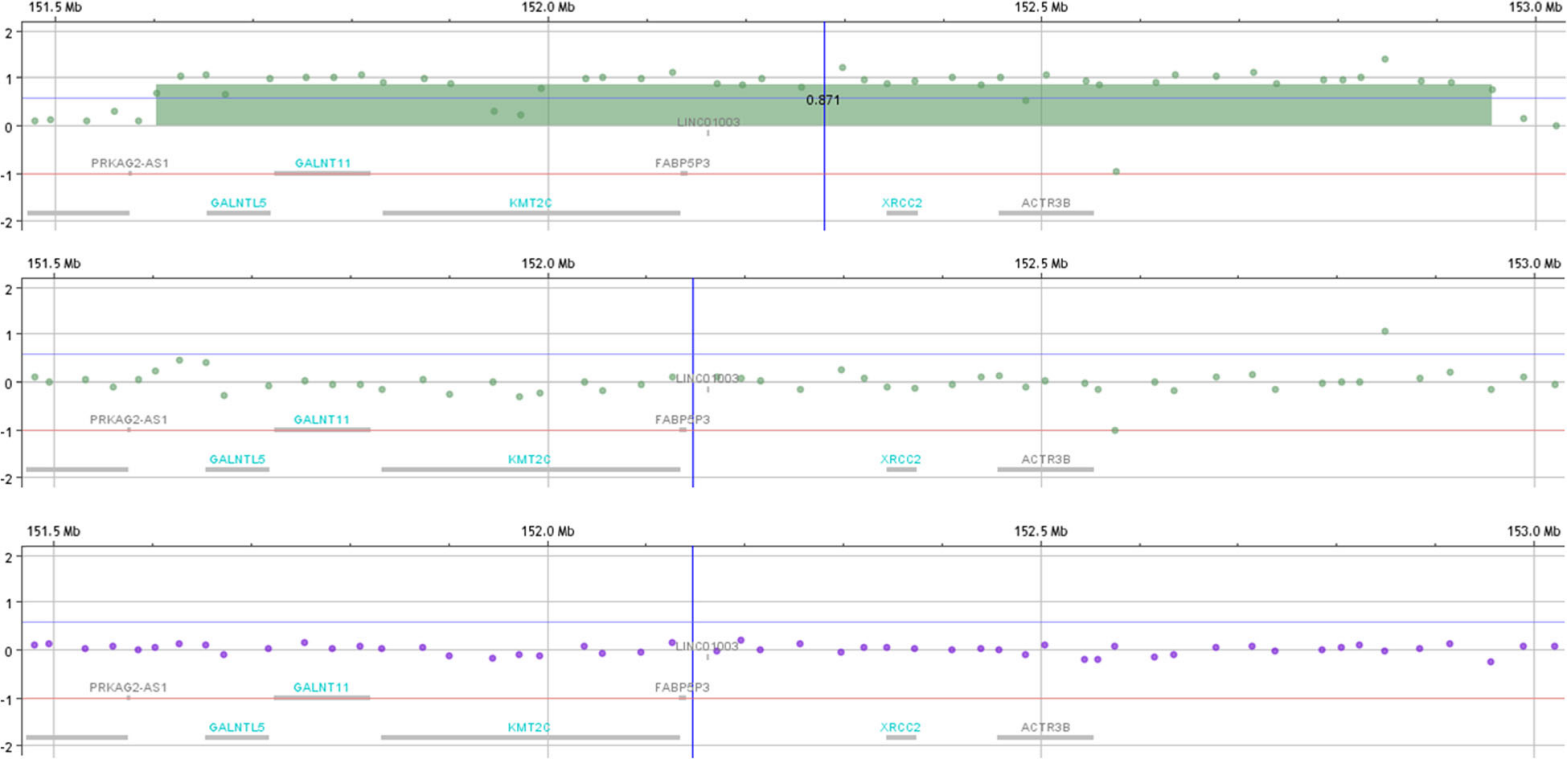
The upper panel shows the triplication at 7q36.1q36.2 encompassing approximately 1.35 Mb in the child. The triplicated region included 77 probes. Five genes (*GALNTL5, GALNT11, KMT2C, XRCC2*, and *ACTR3B* are included in the triplicated region. The other two panels reveal the same region in the mother (middle) and father (bottom) showing normal dosage indicating that the triplication is *de novo* in the child. The test used was chromosomal microarray (CMA) via array comparative genomic hybridization (CGH) which compares a patient’s genomic DNA with a gender-matched reference genomic DNA to detect small copy number gains (duplications) and losses (deletions) on all 46 chromosomes in a single test. PreventionGenetics’ CMA contains ~110,000 distinct CGH probes distributed across the entire genome with a median probe spacing of ~25 kb, and ~59,000 single nucleotide polymorphism (SNP) probes. The CGH probes consist of the entire ISCA (International Standards for Cytogenomic Arrays) Consortium 8x60K version probe set and an additional 60,000 backbone probes (Agilent Technologies, Santa Clara, CA). This includes high-density coverage of ~500 targeted regions with the spacing of 5 kb per probe or at least 20 probes per gene region. These targeted regions include telomere and unique centromere FISH clone regions, microdeletion/duplication regions, genes of known haploinsufficiency, and X-linked mental retardation regions

Chromosomal microarray did identify an ~1.35 Mb triplication at 7q36.1q36.2, corresponding to a minimum triplication boundary of chr7:151,602,419-152,956,632 (hg19). Testing both parents confirmed that this triplication was *de novo* in child (Fig. 2). The CARE guidelines were followed in reporting this case.

## Discussion and conclusions

Herein, we describe a 7q36.1q36.2 triplication in a boy with developmental delay, growth failure (microcephaly, short stature, and underweight), cardiovascular malformation (patent ductus arteriosus, branch pulmonary artery narrowing, and small aortic arch and descending aorta), sensorineural hearing loss, eye manifestations (ptosis, myopia, and astigmatism), genital anomalies (cryptorchidism, hypospadias, and microphallus), lower extremity length discrepancy, bifid uvula, single palmer creases, distinctive facial features (straight eyebrows, ptosis, up-slanted palpebral fissures, broad nasal bridge, low-set and posteriorly rotated ears, small mouth with thick lower lip, microretrognathia, and high-arched palate), prenatal complications (oligohydramnios, IUGR, and decreased fetal movement), and hypotonia, respiratory distress, and feeding difficulty observed in the neonatal period.

The 7q36 triplication in this child, which was found to be *de novo*, has not been previously reported. It is possible that increased dosage of one or more genes in the triplicated region is responsible for the observed phenotype. This region harbors 5 genes: *GALNTL5*, *GALNT11*, *KMT2C*, *XRCC2*, and *ACTR3B*. *GALNTL5* and *GALNT11* encode membrane-bound polypeptide N-acetylgalactosaminyltransferases. These enzymes catalyze O-glycosylation of peptides in the Golgi apparatus [8]. *KMT2C* encodes a histone methyltransferase that can play a role in epigenetic transcriptional activation [9]. *XRCC2* encodes a protein involved in homologous recombination repair of DNA damage. Biallelic mutations in this gene have been associated with Fanconi anemia, complementation group U [10]. *ACTR3B* encodes an actin-related protein that may play a role in the organization of actin cytoskeleton. It is highly expressed in brain, heart, and white blood cells [11].

Among these five genes, increased dosage of *KMT2C* and *ACTR3B* may be proposed to alter transcription and affect cytoskeleton, respectively, causing the pathogenicity of this triplication. However, we suggest that *GALNT11* has the highest potential to be the candidate gene responsible for the observed phenotype because it was shown that the enzyme encoded by this gene can O-glycosylates NOTCH1 leading to the activation of the Notch signaling pathway [12]. The Notch signaling pathway is a highly conserved cellular signaling system that plays a crucial role in metazoan development as it dictates cell fate through regulating differentiation, proliferation, and apoptosis, therefore influencing organ formation and morphogenesis. In mammals, the regulation of neurogenesis, myogenesis, angiogenesis, hematopoiesis, and epithelial-mesenchymal transition are all crucially influenced by Notch signaling. Defects in this pathway has been linked to a diverse group of human diseases including Alagille syndrome, Hajdu–Cheney syndrome, spondylocostal dysostoses type 1, CADASIL (cerebral arteriopathy with subcortical infarcts and leukoencephalopathy), and Alzheimer disease type 3. Notch signaling is also widely implicated in somatic genomic mutational events leading to cancer and malignancy [13]. Therefore, increased *GALNT11* dosage can potentially alter the Notch signaling pathway resulting in a widespread effects and multi-organ manifestations observed in this child with 7q36 triplication.

Although this triplication was found to be *de novo* and includes potential dosage-sensitive genes, it has not been previously described. Therefore, studying further cases with similar genomic rearrangements is needed before making final conclusions about the pathogenicity of this triplication. The likely mechanism for this triplication is non-allelic homologous recombination (NAHR) mediated by segmental duplications. Segmental duplications are estimated to comprise approximately 5% of the human genome and are predicted to facilitate the formation of deletions and duplications through unequal recombination between homologous regions [14]. The region of chromosome 7 involved in this case (7q36) has been reported to be flanked by segmental duplications that could mediate the formation of chromosomal rearrangements [15].

In summary, we present a boy with developmental delay, growth failure, cardiovascular malformation, sensorineural hearing loss, eye manifestations, genital anomalies, lower extremity length discrepancy, bifid uvula, and distinctive facial features who was found to have an ~1.35 Mb triplication at 7q36.1q36.2. The triplicated region includes *GALNTL5*, *GALNT11*, *KMT2C*, *XRCC2*, and *ACTR3B*. *GALNT11* encodes a membrane-bound polypeptide N-acetylgalactosaminyltransferase that can O-glycosylate NOTCH1 leading to the activation of the Notch signaling pathway. Therefore, increased *GALNT11* dosage can potentially alter the Notch signaling pathway supporting the pathogenicity of 7q36 triplication.
